# Impact of Mitochondrial Ca^2+^-Sensitive Potassium (mBK_Ca_) Channels in Sildenafil-Induced Cardioprotection in Rats

**DOI:** 10.1371/journal.pone.0144737

**Published:** 2015-12-15

**Authors:** Friederike Behmenburg, Marianne Dorsch, Ragnar Huhn, David Mally, André Heinen, Markus W. Hollmann, Marc M. Berger

**Affiliations:** 1 Department of Anesthesiology, University Hospital Düsseldorf, Düsseldorf, Germany; 2 Department of Cardiovascular Physiology, Heinrich-Heine University Düsseldorf, Düsseldorf, Germany; 3 Department of Anesthesiology, Laboratory of Experimental Intensive Care and Anesthesiology (L.E.I.C.A.), Academic Medical Center (AMC), University of Amsterdam, Amsterdam, The Netherlands; 4 Department of Anesthesiology, Perioperative and General Critical Care Medicine, Salzburg General Hospital, Paracelsus Medical University, Salzburg, Austria; Emory University, UNITED STATES

## Abstract

**Background:**

Mitochondrial large-conductance Ca^2+^-sensitive potassium (mBK_Ca_) channels are involved in myocardial ischemic preconditioning. Their role in sildenafil-induced cardioprotection is unknown. We investigated whether sildenafil-induced acute cardioprotection is mediated by activation of mBK_Ca_ channels in the rat heart *in vitro*.

**Methods:**

Male Wistar rats (n = 8 per group) were randomized and anesthetized with pentobarbital (90 mg/kg). Hearts were isolated, mounted on a Langendorff system and perfused with Krebs-Henseleit buffer at a constant pressure of 80 mmHg. Hearts underwent 30 min of global ischemia followed by 60 min of reperfusion. At the end of the experiments infarct size was determined by TTC staining. In the control group rats were not further treated. Sildenafil (3 μM) was administered over 10 min before the beginning of ischemia. The mBK_Ca_ channel inhibitor paxilline (1 μM) was administered with and without sildenafil before the onset of ischemia. The pathway underlying sildenafil-induced cardioprotection was further investigated with the protein kinase G blocker KT5823 (1 μM). Myocardial cGMP concentration was measured by ELISA. Data (mean±SD) were analysed with a one and two-way analysis of variance as appropriate.

**Results:**

In control animals infarct size was 52±8%. Sildenafil increased cGMP concentration and reduced infarct size to 35±6% (P<0.05 vs. control). Paxilline and KT5823 completely blocked sildenafil-induced cardioprotection (paxilline+sildenafil: 50±8%, KT5823+sildenafil: 45±8%; both P<0.05 vs. sildenafil). Functional heart parameters and coronary flow were not different between the study groups.

**Conclusion:**

This study shows that in male rats protein kinase G-dependent opening of mBK_Ca_ channels plays a pivotal role in sildenafil-induced cardioprotection.

## Introduction

Ischemic heart disease is the leading cause of death worldwide, accounting for about 11% of all deaths globally [1]. After an acute myocardial infarction early reperfusion is the most important therapy for improving morbidity and mortality. However, reperfusion itself induces injury that accounts for up to 50% of the final size of a myocardial infarction and paradoxically reduces the beneficial effects of a restored coronary blood flow [2]. Since the end of the last century much effort has, therefore, been focused on developing cardioprotective strategies that render the heart more resistant to ischemia-reperfusion injury.

Sildenafil is a phosphodiesterase-5-inhibitor that was first approved for the treatment of erectile dysfunction in 1998 [3], and approved for the treatment of pulmonary arterial hypertension in 2005 [4]. The effectiveness of sildenafil under these conditions is attributed to its vasodilating and anti-proliferative properties. The vasodilating effect is mediated by inhibitition of the breakdown of cyclic guanosine monophosphate (cGMP), which relaxes smooth muscle cells [5], whereas the anti-proliferative effect involves protein kinase A and protein kinase G (PKG) activated pathways [6]. Several animal studies suggest that sildenafil also protects against myocardial ischemia-reperfusion injury and thereby reduces infarct size by about 35–75% relative to untreated conditions [5,7–13]. The cardioprotective effects of sildenafil are thought to be mediated by opening of mitochondrial ATP-sensitive potassium (mito-K_ATP_) channels [14], enhanced expression of endothelial nitric oxide synthase (NOS) and inducible NOS [5], and activation of protein kinase C and PKG [15]. However, the precise molecular mechanisms and signalling pathways underlying the cardioprotective effects remain to be determined.

The present study investigated whether sildenafil-induced acute cardioprotection in rats involves large-conductance Ca^2+^-sensitive potassium (BK_Ca_) channels. In cardiomyocytes BK_Ca_ channels are located on the inner membrane of mitochondria [16], so that they constitute mBK_Ca_ channels, which have previously been described by us and others to be a critical downstream target in the signalling pathway of several cardioprotective interventions [17–22]. These channels, i.e. mBK_Ca_ channels, are in part regulated by PKG and thus share a common signalling pathway with sildenafil [23,24]. Unravelling the signalling pathway underlying the sildenafil-induced cardioprotection may provide a conceptual framework for developing novel, more specific cardioprotective strategies.

## Methods

The current investigation was conducted in accordance with the *Guide for the Care and Use of Laboratory Animals* published by the National Institutes of Health (Publication number 85–23, revised 1996) and was performed after obtaining approval from the Animal Ethics Committee of the University of Düsseldorf, Germany. Seventy-six male Wistar rats (Charles River), weighing 285±23 g were housed on a 12:12 light/dark schedule with free access to standard chow and water. All chemicals were purchased from Sigma-Aldrich (Taufkirchen, Germany).

### Surgical preparation

Surgical preparation was performed as described previously [18]. In brief, rats were anesthetized by intraperitoneal injection of pentobarbital (90 mg/kg). After thoracotomy hearts were excised and mounted on a Langendorff system. Perfusion of the hearts was performed at constant pressure (80 mmHg) with a Krebs-Henseleit solution, containing (in mM): 116 NaCl, 4.7 KCl, 1.1 MgSO_4_, 1.17 KH_2_PO_4_, 24.9 NaHCO_3_, 2.52 CaCl_2_, 8.3 glucose, and 2.2 pyruvate at 37°C. The perfusate was bubbled with a mix of 95% O_2_ and 5% CO_2_, resulting in a pO_2_ of 540–620 mmHg, a pCO_2_ of 35–38 mmHg, and a pH of 7.38–7.43, respectively.

A fluid filled balloon was inserted into the left ventricle and end-diastolic pressure was set at 1–4 mmHg. All hearts underwent an equilibration period of 20 minutes. Thereafter, heart rate, the rate pressure product (RPP, calculated as heart rate x (maximal left ventricular pressure—minimal left ventricular pressure)), left ventricular end-diastolic pressure (LVEDP), and coronary flow were measured continuously and digitized using an analogue to digital converter (PowerLab/8SP, ADInstruments Pty Ltd, Castle Hill, Australia) at a sampling rate of 500 Hz. The data were continuously recorded on a personal computer using Chart for Windows v5.0 (ADInstruments Pty Ltd, Castle Hill, Australia). Maximal contracture and time to maximal contracture were detected by checking the course of contracture development during index ischemia and selecting the time point when contracture reached its highest level in each experiment. Arrhythmic intervals were not used for data analysis.

#### Experimental protocol

**Fig 1:**
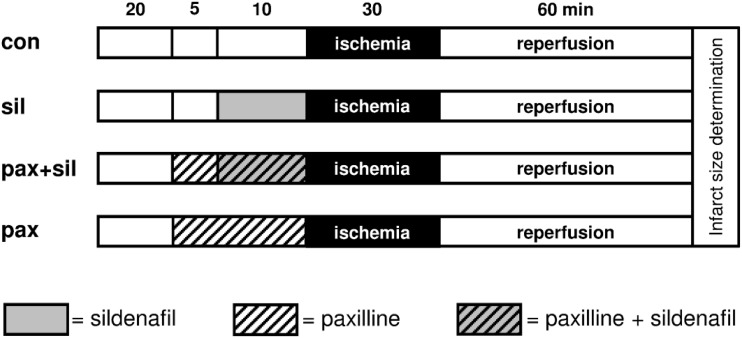
Experimental design of the 1st part of the study: con = control, sil = sildenafil, pax = paxilline. After a 20 minutes equilibration period paxilline (1 μM) was administered to group 3 and group 4. In group 2 and 3 sildenafil (3 μM) was administered 10 minutes before global myocardial ischemia was induced.

**Fig 2:**
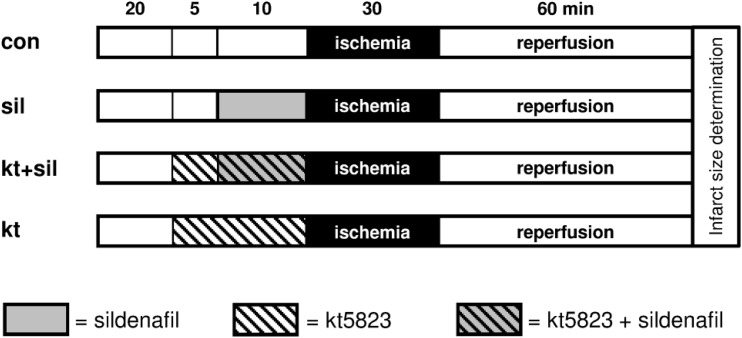
Experimental design of the 2nd part of the study to investigate the role of protein kinase G (PKG) in sildenafil-induced cardioprotection.: con = control, sil = sildenafil, kt = KT5823 (PKG blocker).

Figs 1 and 2 summarize the study design. In the first part of the study (Fig 1) hearts were randomly assigned to one of four experimental groups (n = 8 in each group). In group 1 (control) hearts were kept under baseline conditions for 35 minutes before they underwent 30 minutes of ischemia followed by 60 minutes of reperfusion. Group 2 (sildenafil) was designed to investigate the cardioprotective effect of sildenafil. For this purpose, 3 μM sildenafil were given over 10 minutes prior to ischemia. Group 3 (paxilline+sildenafil) was designed in order to characterize the role of mBK_Ca_ channels in the signalling cascade underlying sildenafil-induced cardioprotection. In this group the potent and well established [18,22,25,26] BK_Ca_ channel inhibitor paxilline (1 μM) was given 5 minutes before sildenafil was administered. To rule out an effect on myocardial infarction size by paxilline itself, paxilline (1 μM) was also administered without sildenafil (group 4, paxilline). Sildenafil and paxilline were each separately infused into a mixing chamber placed in the perfusion system. After 60 minutes of reperfusion, the hearts were cut into transverse slices, which were then stained with 0.75% triphenyltetrazoliumchloride (TTC) solution. The infarcted area was determined by planimetry using SigmaScan Pro 5^®^ computer software (SPSS Science Software, Chicago, IL).

In the second part of the study (Fig 2) the effect of the selective PKG inhibitor KT5823 on sildenafil-induced cardioprotection was investigated. For this purpose hearts were randomized to four experimental groups (n = 8 in each group). Group 1 (control) and group 2 (sildenafil) underwent the same protocol as described for these groups above. In group 3 KT5823 (1 μM) was given 5 minutes before sildenafil was administered. To rule out an effect of KT5823 on myocardial infarction size, KT5823 (1 μM) was also administered without sildenafil (group 4). After 60 minutes of reperfusion the infarct size was determined as described above.

In a subset of experiments myocardial cGMP concentration was measured in both control animals (n = 6) and in animals exposed to sildenafil (n = 6). These measurements were performed after administration of sildenafil was terminated, i.e. directly before ischemia was induced. cGMP concentration was measured by ELISA as described previously [27].

### Statistical analysis

Sample size was calculated using GraphPad StatMate^™^ Version 1.01 (GraphPad Software, San Diego, CA, USA), and yielded a group size of n = 8 as necessary to detect a difference in infarct size of 25% with a power of 80% and an α < 0.05 (two-tailed). The estimations of the mean difference of 25% and the standard deviation of 15% were based on previous own data [18]. Hemodynamic parameters were measured during baseline, and during the ischemic and reperfusion period. Comparisons of hemodynamics between groups or between different time points within a group were performed with a two-way analysis of variance (ANOVA) followed by Tukey’s post hoc test (SPSS Science Software, version 12.0.1). Infarct sizes were determined by a researcher blinded to the experimental groups and analyzed by One-way ANOVA followed by Student Newman-Keuls post hoc test. Data are expressed as mean±SD. Changes within and among groups were considered statistically significant if *P*<0.05.

## Results

### Infarct size

**Fig 3:**
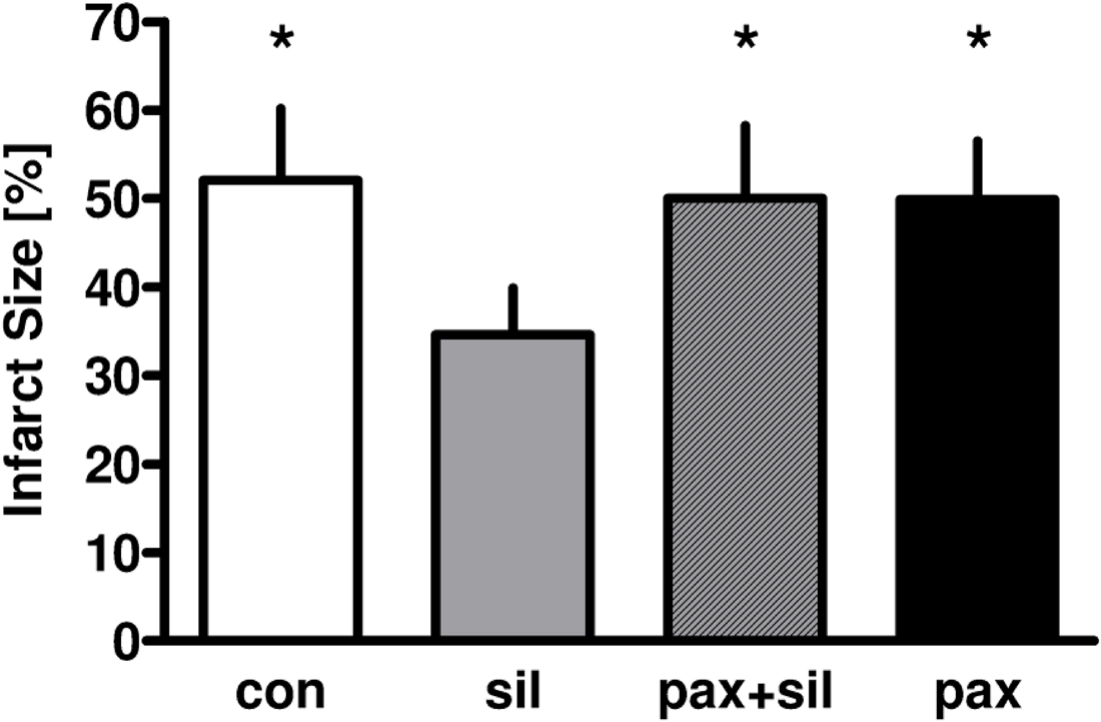
Infarct size of untreated control hearts (con), and of hearts exposed to sildenafil (sil, 3 μM) with or without the mBK_Ca_-channel inhibitor paxilline (pax, 1 μM).

In the first part of the study infarct size of the control group was 52±8% of the whole heart (Fig 3). Sildenafil reduced infarct size to 35±6% (P<0.05 versus control). When paxilline was administered prior to sildenafil, the infarct size was about the same as in untreated controls (50±8%, P<0.05 versus sildenafil alone), indicating that the BK_Ca_-channel inhibitor paxilline completely blocked the cardioprotective effect of sildenafil. Paxilline alone hat no effect on infarct size (50±7%, P<0.05 versus sildenafil alone).

**Fig 4:**
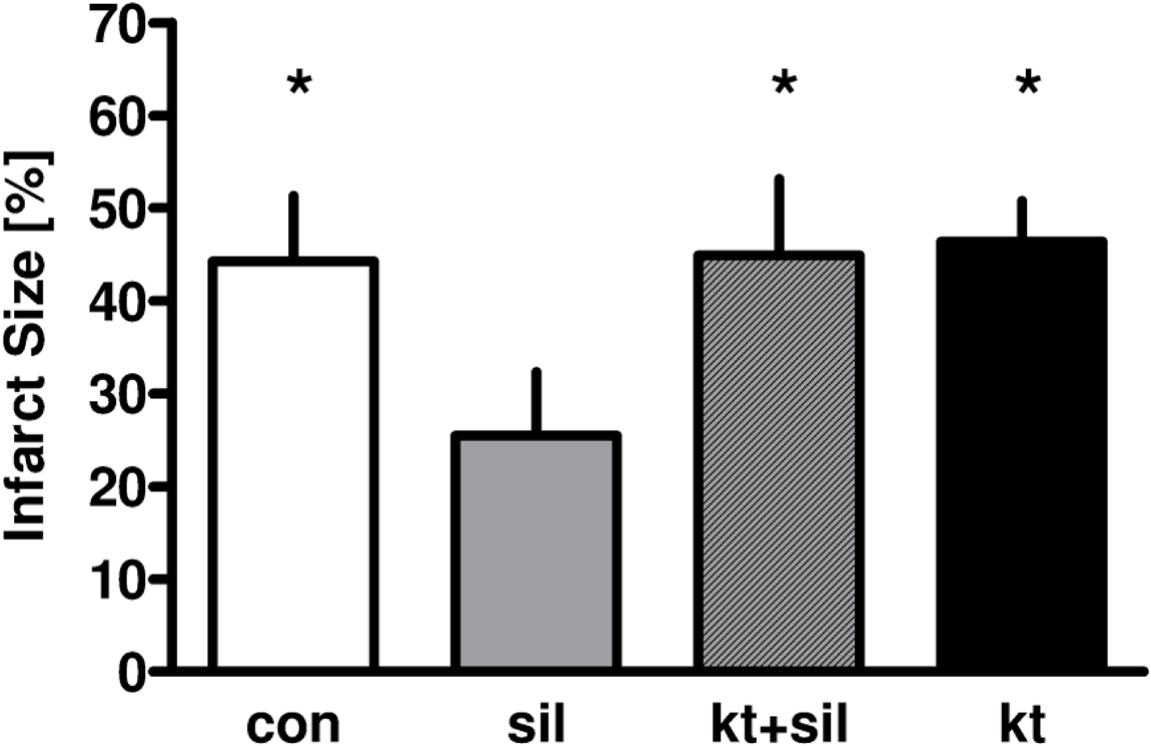
Infarct size in the 2^nd^ part of the study that investigated the effect of the PKG-blocker KT5823 on infarct size. Data are mean±SD. *P<0.05 versus sildenafil.

In the second part of the study, which was designed to investigate the effect of the PKG inhibitor KT5823, the infarct size of the control group was 44±7% (Fig 4). Sildenafil decreased infarct size to 26±7% (P<0.05 versus control), and this effect was completely blocked by KT5823 (infract size 45±8%, P<0.05 versus sildenafil alone). KT5823 alone had no effect on infarct size (46±4%, P<0.05 versus sildenafil alone).

### cGMP formation

**Fig 5:**
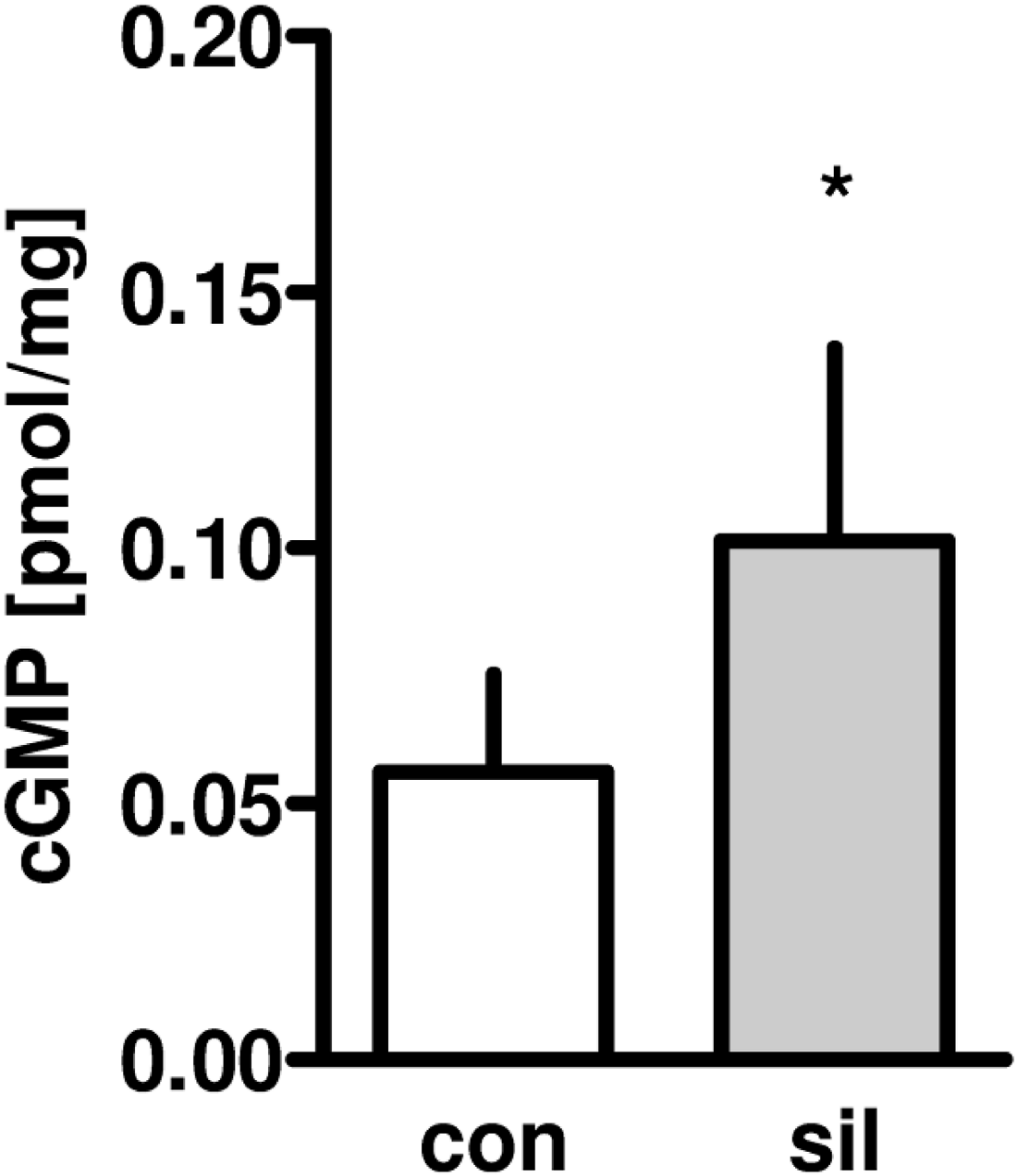
Effect of sildenafil on myocardial cGMP concentration (n = 6 per group).

As shown in Fig 5, sildenafil lead to a significant increase in myocardial cGMP formation compared to untreated controls (P<0.05).

### Cardiac function

As summarized in Table 1 (first part of the study) and Table 2 (second part of the study), at none of the different time points was there a significant difference in the heart rate, RPP, LVEDP, and coronary blood flow between the different experimental groups. Over the duration of the experiments heart rate remained stable in all groups, whereas RPP and LVEDP increased during the reperfusion period (all P<0.05 versus baseline). In contrast, coronary flow decreased during the reperfusion period (P<0.05 versus baseline).

**Table 1:** Hemodynamic variables.

	baseline	drug perfusion	ischemia	reperfusion	
			30	30	60
*Heart Rate (bpm)*					
con	326 ± 29	330 ± 43		293 ± 36	275 ± 24
sil	305 ± 28	336 ± 20		330 ± 39	278 ± 26
pax+sil	308 ± 58	308 ± 57		264 ± 50	277 ± 41
pax	338 ± 50	334 ± 53		296 ± 50	275 ± 37
*RPP (mmHg* * *bpm)*					
con	42704 ± 6340	42704 ± 6340		7940 ± 4844*	9544 ± 3368*
sil	43200 ± 9234	46046 ± 6831		8662 ± 5223*	9972 ± 2840*
pax+sil	45168 ± 9736	43034 ± 8034		7817 ± 4875*	10135 ± 2852*
pax	44487 ± 6362	40855 ± 7904		6873 ± 2347*	8018 ± 2185*
*LVEDP (mmHg)*					
con	7 ± 3	8 ± 3	39 ± 9*	85 ± 15*	78 ± 13*
sil	8 ± 1	10 ± 1	33 ± 6*	87 ± 13*	80 ± 11*
pax+sil	9 ± 4	11 ± 3	36 ± 4*	84 ± 8*	77 ± 10*
pax	10 ± 5	14 ± 6	40 ± 12*	89 ± 15*	82 ± 15*
*Coronary flow (ml* * *min* ^*-1*^ */g dry heart weight)*					
con	105 ± 15	113 ± 17		66 ± 15*	57 ± 12*
sil	107 ± 10	117 ± 9		60 ± 15*	55 ± 17*
pax+sil	114 ± 16	114 ± 19		51 ± 22*	51 ± 5*
pax	112 ± 13	103 ± 13		61 ± 11*	53 ± 8*

Data are mean±SD. con = control; sil = sildenafil; pax = paxilline. RPP = rate pressure product; LVEDP = left ventricular end-diastolic pressure.

*P<0.05 versus baseline.

**Table 2:** Hemodynamic variables.

	baseline	drug perfusion	ischemia	reperfusion	
			30	30	60
*Heart Rate (bpm)*					
con	334 ± 37	316 ± 36		238 ± 43*	249 ± 41*
sil	295 ± 40	305 ± 43		226 ± 117	244 ± 39
kt+sil	313 ± 57	306 ± 54		198 ± 109*	252 ± 56
kt	317 ± 32	298 ± 36		287 ± 48	256 ± 34
*RPP (mmHg* * *bpm)*					
con	38004 ± 6029	35803 ± 6717		6021 ± 3845*	7840 ± 3220*
sil	34296 ± 4887	35525 ± 4167		4036 ± 2255*	5964 ± 2297*
kt+sil	36083 ± 6829	32697 ± 4745		6403 ± 5231*	6076 ± 2809*
kt	37895 ± 6834	33894 ± 3521		6300 ± 4834*	6736 ± 3338*
*LVEDP (mmHg)*					
con	4 ± 2	5 ± 4	32 ± 13*	72 ± 12*	66 ± 9*
sil	5 ± 2	7 ± 3	30 ± 6*	70 ± 9*	64 ± 8*
kt+sil	4 ± 2	6 ± 3	31 ± 8*	64 ± 12*	60 ± 9*
kt	4 ± 2	6 ± 3	31 ± 9*	77 ± 10*	70 ± 8*
*Coronary flow (ml* * *min* ^*-1*^ */g dry heart weight)*					
con	101 ± 15	100 ± 14		59 ± 5*	56 ± 3*
sil	95 ± 22	100 ± 21		66 ± 15*	59 ± 13*
kt+sil	100 ± 21	100 ± 21		61 ± 11*	57 ± 14*
kt	92 ± 20	89 ± 23		62 ± 6*	59 ± 7*

Data are mean±SD. con = control; sil = sildenafil; kt = kt5823. RPP = rate pressure product; LVEDP = left ventricular end-diastolic pressure.

*P<0.05 versus baseline.

### Animal characteristics

There were no differences in body weight or heart weight, respectively, between the different experimental groups (Tables 3 and 4).

**Table 3:** Weights and ischemic contracture.

	Body weight (g)	Heart weight dry (g)	Time of max. ischemic contracture (min)	Level of max. ischemic contracture (mmHg)
con	279 ± 22	0.14 ± 0.02	15 ± 1	66 ± 12
sil	284 ± 23	0.15 ± 0.02	15 ± 1	59 ± 9
pax+sil	285 ± 19	0.15 ± 0.01	16 ± 1	60 ± 11
pax	287 ± 29	0.15 ± 0.01	15 ± 1	66 ± 18

Data are mean±SD. con = control; sil = sildenafil; pax = paxilline.

**Table 4:** Weights and ischemic contracture.

	Body weight (g)	Heart weight dry (g)	Time of max. ischemic contracture (min)	Level of max. ischemic contracture (mmHg)
con	270 ± 16	0.16 ± 0.01	15 ± 1	48 ± 14
sil	291 ± 21	0.16 ± 0.02	15 ± 2	50 ± 6
kt+sil	295 ± 32	0.16 ± 0.02	15 ± 2	46 ± 12
kt	264 ± 19	0.16 ± 0.01	16 ± 1	47 ± 9

Data are mean±SD. con = control; sil = sildenafil; kt = kt5823.

## Discussion

Over the past 30 years enormous progress has been made in understanding the mechanisms underlying myocardial ischemia-reperfusion injury, and has led to the development of several experimental cardioprotective strategies. Despite their unquestionable efficacy in these settings, however, only few of them have been integrated into clinical pathways yet. This may be in part due to the lack of safe and effective drugs with an acceptable side effect profile that exert powerful cardioprotection.

### Sildenafil-induced acute cardioprotection

The present study shows that the phoshphodiesterase-5-inhibitor sildenafil has a powerful acute cardioprotective effect by reducing ischemia-reperfusion injury of the isolated perfused rat heart subjected to 30 minutes of global ischemia and 60 minutes of reperfusion in the Langendorff model, which is in line with previous studies, e.g. [7,12,13]. With regard to the clinical situation this finding might be of particular interest: first, sildenafil is a drug with a desirable side effect profile that is widely used for the treatment of erectile dysfunction and pulmonary arterial hypertension [14]. Its pharmacokinetic and pharmacodynamic profile is well described and the drug is widely available. Second, measures that acutely reduce the extent of an ischemia-reperfusion injury can be administered just before a planned coronary occlusion or before coronary reperfusion occurs, e.g. in coronary bypass surgery, what usually is a realizable approach in the clinical setting. Of note, and in addition to its infarct size reducing effect, administration of sildenafil before coronary occlusion has previously been shown to reduce cardiac hypertrophy and apoptosis [28], to improve coronary vascular resistance and hemodynamics [29], and to improve survival [28] in several experimental animal models. The sildenafil dose (3 μM) used in the present study is in the same range as in previous studies investigating the beneficial effect of sildenafil on erectile dysfunction and on lower urinary tract symptoms [30–32].

The use of sildenafil for acute cardioprotection in the setting of ischemia-reperfusion appears promising, and unravelling the underlying mechanisms is an attractive strategy for developing more specific clinical interventions capable of protecting an ischemic myocardium against an ischemia-reperfusion injury. However, since the present study was performed on male rats only, the findings are not necessarily applicable to females.

### Signalling pathway in sildenafil-induced acute cardioprotection

**Fig 6:**
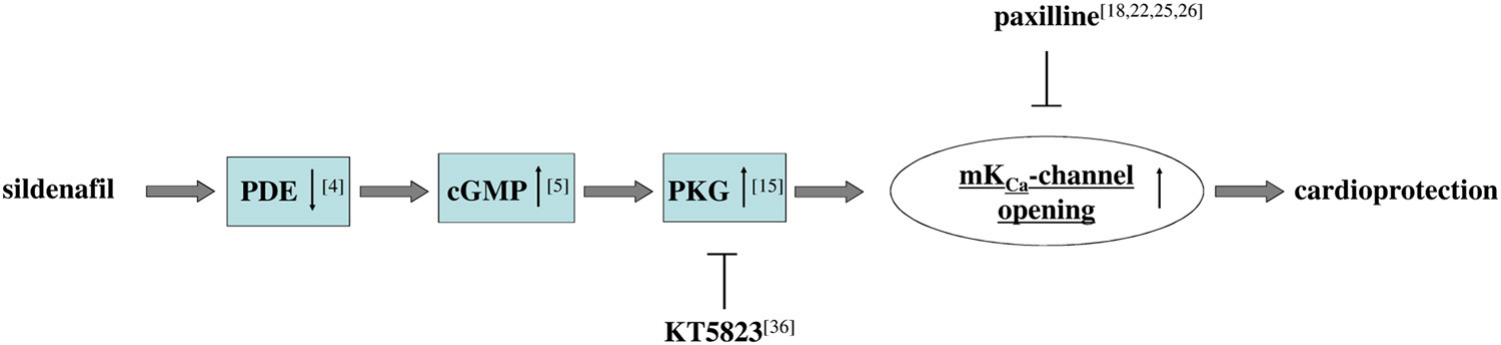
Schematic model of the cardioprotective pathway of sildenafil-induced acute cardioprotection as reflected by the present study. PDE = phosphodiesterase, PKG = protein kinase G, KT5823 = protein kinase G inhibitor, Paxilline = mBK_Ca_-channel inhibitor.

The results of the present study show that sildenafil-induced acute cardioprotection was completely abolished when paxilline, a potent and selective BK_Ca_ channel blocker [18,22,25,26], was administered before sildenafil was injected, while paxilline itself had no effect on infarct size. Because in cardiomyocytes BK_Ca_ channels are located on the inner mitochondrial membrane and not in other subcellular structures [19], this finding suggests that opening of mitochondrial BK_Ca_ (mBK_Ca_) channels is essential in the early cardioprotective effect of sildenafil. As described previously, mitochondria play a pivotal role in controlling cell life and death by ATP synthesis and Ca^2+^ homeostasis, and thus in ischemia-reperfusion injury [7]. We and others previously found mBK_Ca_ channels to be a critical downstream target in the signalling pathway of several cardioprotective interventions [17–22]. These channels are members of the voltage-gated K^+^ channel superfamily and are critical in maintaining Ca^2+^ homeostasis, mainly via their ability to sense transmembrane voltage and intracellular Ca^2+^ concentration [33]. mBK_Ca_ channels are in part regulated by PKG and thus share a common signalling pathway with sildenafil [15,23,24,34]. Therefore it appeared possible that sildenafil-induced cardioprotection is PKG-dependent. Indeed, our results show that administration of the selective PKG inhibitor KT5823 completely blocked the cardioprotective effect of sildenafil, suggesting that PKG plays a crucial role in the signalling cascade underlying sildenafil-induced cardioprotection as illustrated in Fig 6. This finding is supported by the observation that the selective knockdown of protein kinase G in cardiomyocytes has been shown to block sildenafil-induced cardioprotection [33]. Because sildenafil also increased cGMP formation the results of the present study indicate that sildenafil-induced cardioprotection is mediated, at least in part, by enhanced cGMP synthesis and PKG activation, which, in turn, opens myocardial mBK_Ca_ channels.

### Effects of sildenafil on cardiac function

Intravenous administration of sildenafil over 10 minutes prior to ischemia did not alter cardiac function. While heart rate remained stable throughout the whole duration of the experiments, RPP decreased and LVEDP increased upon reperfusion without differences between the treatment groups. These findings indicate that sildenafil did not affect cardiac performance, e. g. cardiac contractility, which is in line with previous observations, e.g. [13]. In addition, coronary flow was significantly reduced after ischemia and was not affected by sildenafil, suggesting that the acute cardioprotective effect of sildenafil occurred independent of potential vasodilating effects in the coronary vasculature. Why coronary flow was not increased in response to sildenafil remains, however, unclear.

### Limitations

In the present study the BK_Ca_ channel blocker paxilline was used to characterize the signaling pathway underlying sildenafil-induced cardioprotection. Paxilline is a potent and well established blocker of various BK_Ca_ channels [18,22,25,26]. Therefore, its exclusive use as a pharmacological indicator of the function of mitochondrial BK_Ca_ channels needs to be taken with caution. Several studies suggest that BK_Ca_ channels in cardiomyocytes are exclusively located on the inner mitochondrial membrane, and not in other subcellular structures [16,35–38], indicating that the paxilline results of the present study reflect the role of mBK_Ca_ channels in sildenafil-induced cardioprotection. This interpretation is in line with the observation that pharmacological activation of mitochondrial BK_Ca_ channels has previously been shown to protect isolated cardiomyocytes against reperfusion injury [35]. However, a recent study by Lai et al. [39] found BK_Ca_ channels to be located in plasma membranes of sinoatrial node cells, so that in the heart the expression of BK_Ca_ channels outside of mitochondria cannot conlusively be ruled out.

The present study did not investigate gender-dependent differences and instead focused on male animals as most of the studies investigating sildenafil-induced cardioprotection did. It thus remains unclear whether the results are applicable to female rats. It also remains unclear whether the results are transferrable to multi-morbid patients. Clinical studies investigating patient populations with an according diseases profile are needed to address this issue.

### Conclusion

In recent years, several studies investigated the role and mechanisms of sildenafil in protecting against ischemia-reperfusion injury. These studies identified the expression of nitric oxide synthases, accumulation of cGMP, activation of kinases (such as PKC and ERK), and opening of mitochondrial K_ATP_ channels as part of the signalling cascade. The present study investigated the acute cardioprotective effect of sildenafil and found that sildenafil-induced cardioprotection is mediated, at least in part, by enhanced cGMP synthesis and PKG activation, which, in turn, opens myocardial mBK_Ca_ channels (Fig 6).
